# *Everything is Tuberculosis*: A Portrait of Connection

**DOI:** 10.3201/eid3203.260264

**Published:** 2026-03

**Authors:** Nkuchia M. M’ikanatha

**Affiliations:** Center for Clinical Epidemiology and Biostatistics, Perelman School of Medicine, University of Pennsylvania, Philadelphia, Pennsylvania, USA; The Pennsylvania State University, University Park, Pennsylvania, USA

**Keywords:** tuberculosis and other mycobacteria, bacteria, respiratory infections, antimicrobial resistance, Sierra Leone, John Green, history of medicine

In 2019, author John Green visited Sierra Leone as a volunteer for Partners in Health, an international nonprofit organization, to learn about the maternal and neonatal healthcare system. While touring Lakka Government Hospital in Freetown, Sierra Leone, Green met Henry Reider, a 17-year-old tuberculosis (TB) patient. Green at first mistook him for a child—Henry’s stature had been stunted by drug-resistant TB. Green writes:

I figured Henry was someone’s kid—a doctor, maybe, or a nurse, or one of the cooking or cleaning staff. Everyone seemed to know him, and everyone stopped their work to say hello and rub his head or squeeze his hand. I was immediately charmed by Henry—he had some of the mannerisms of my son, the same paradoxical mixture of shyness and enthusiastic desire for connection.

The two struck up an acquaintance, and the relationship opened up a world Green didn’t know existed. *Everything is Tuberculosis*, the book that grew out of Green’s experience with Henry, is part cultural history, part a meditation on friendship, and finally a call to action for effective and equally distributed treatment for this disease.

TB, Green writes, is one of the world’s oldest diseases; signs of infection have been detected in human fossil remains. Before the modern era, many TB patients literally wasted away, just like Henry. Historically, shifting names for TB have reflected cultural attempts to characterize its devastating effects. The Chinese word was *huifu* (destroyed palace), in Hebrew the disease was described as *schachepheth* (wasting away), in Greek the name was *pththisis* (to decay), and in English “consumption” gained favor in the 1800s. Today, we take the name from the bacillus that causes the disease, *Mycobacterium tuberculosis*, identified and described by Robert Koch in 1882.

TB progression can seem random; a person may have an active case, then quiescence, and then relapse into another bout of active symptoms. Beyond the biological challenges, Green describes how cultural attitudes, deep-seated biases, and commercial incentives continue to complicate global treatment and prevention efforts.

After meeting Henry Reider and hearing his story, the author began to understand the enormity and complexity of TB and the factors that contribute to its persistence. He had thought of the disease, he writes, as “a disease of 19th century poets,” but after becoming acquainted with Henry, Green began to see TB as “… both a form and expression of injustice.”

Green notes that, in the 19th Century, the disease was strangely associated with fleeting youth and genius. Lord Byron famously remarked on his own pale appearance, wishing to die of consumption so women would find his “dying look” interesting. Poets like Keats called it a “delicious diligent disease,” and even Henry Gilbert’s 1842 medical treatise contained an ode to the “beauty of female consumptives,” describing a “rosy tint” on the cheeks that was actually the fever of a dying patient:

With step as noiseless as the summer air,

Who comes in beautiful decay? Her eyes

Dissolving with a feverish glow of light;

————————————and on

Her cheek a rosy tint, as if the tip

Of beauty’s finger faintly press’d it there:

Alas! Consumption is her name.

TB has been heavily stigmatized throughout history and continues to be so. Today, TB is often seen as a mark of disgrace because of its association with poverty, but it is also often associated with perceived choice and moral failures.

TB drugs are unavailable to many patients, especially in locations where healthcare is not as well supported, and the treatment requires long-term care involving many doses of antibiotics. In Sierra Leone, clinics are often some distance from towns and villages. The narratives notes, in places the drugs or the means to administer them are not there at all, and in dozens of countries, treatment either wasn’t available or reached patients only sporadically. “It was as if the cure did not exist—because the cure is where the disease is not, and the disease is where the cure is not,” Green writes.

After learning Henry Reider’s story, Green returned to the United States, where he found he “could not shut up about tuberculosis.” What he had seen and heard profoundly changed him. He found himself unable to grasp the enormous reach of tuberculosis and explained the struggle this way:

The problem with statistics is that I cannot take in what it means to lose 1,250,000 people each year to a curable illness… That’s more than a hundred thousand people a month. But how do I conceptualize such statistics? I’ve been in a stadium with a hundred thousand people, but I didn’t know each of their families. I didn’t know about the people they’ve loved, the heartbreaks they’ve endured, their constraints and encouragements, their frailty and resilience. I simply cannot fathom what 1,250,000 means.

The suffering that Henry Reider experienced—because of poverty, geography, and his misfortune of contracting this disease—led Green to consider not only the injustice of his situation but how it could be counteracted. His conclusion, in this highly personal account, is this:

… when we know about suffering, when we are proximal to it, we are capable of extraordinary generosity. We can do and be so much for each other—but only when we see one another in our full humanity, not as statistics or problems, but as people who deserve to be alive in the world.

*Everything is Tuberculosis* is about friendship and how we can become changed—and charged—to pursue solutions to problems. The book is a portrait of connection, in both large and small ways. Green’s portrait of Henry, and their developing closeness, drew him to the complex, colossal world of TB. He writes, “It’s only because I met Henry Reider in 2019 that this book exists, and that I’ve found a hopefully good use for the curious megaphone I lucked into. TB has become the organizing principle of my professional life over the last 5 years.”

The friendship Green forged with Henry serves as a model for how to move from statistics to action. Green argues that global health often fails because we view patients as data points, or part of a cost–benefit analysis, rather than as people. This text quietly straddles intimacy and action, appealing to researchers and the general audience alike. Its personal focus makes the content accessible in ways that raise awareness of TB—a treatable, preventable disease—and the systemic issues that keep it from being resolved. Green shows us we all have a stake in this collective effort.

By the final pages, Henry Rieder has become a close friend—“He and I talk all the time now”—leaving the reader to draw the connection that recognizing our shared humanity is what truly sustains progress in addressing the disease. Before closing the book, Green shows a picture of a beaming Reider in black and white. Reider is resting his elbow on a camera tripod and holds his head while looking at the reader. The men make videos, one in Sierra Leone and the other in the United States, their vision to overcome stigma created by geography and misunderstanding of the other, to ultimately address the pathogen that causes TB in the language microbes respect. As Green says:

Imagining someone as more than human does as much the same work as imagining them as less than human. The ill are treated as fundamentally “other” because the social order is frightened by what their frailty reveals about everyone else’s.

In addition to *M. tuberculosis*, this other, Green says, is what we need to overcome.
